# Coupling alkaline pre-extraction with alkaline-oxidative post-treatment of corn stover to enhance enzymatic hydrolysis and fermentability

**DOI:** 10.1186/1754-6834-7-48

**Published:** 2014-04-03

**Authors:** Tongjun Liu, Daniel L Williams, Sivakumar Pattathil, Muyang Li, Michael G Hahn, David B Hodge

**Affiliations:** 1 DOE-Great Lakes Bioenergy Research Center, Michigan State University, East Lansing, MI, USA; 2 School of Food and Bioengineering, Qilu University of Technology, 250353 Jinan, China; 3 Department of Chemical Engineering and Materials Science, Michigan State University, 48824 East Lansing, MI, USA; 4 Complex Carbohydrate Research Center, The University of Georgia, 315 Riverbend Rd, 30602 Athens, GA, USA; 5 BioEnergy Science Center (BESC), Oak Ridge National Laboratory, 37831 Oak Ridge, TN, USA; 6 Department of Biosystems and Agriculture Engineering, Michigan State University, 48824 East Lansing, MI, USA; 7 Department of Plant Biology, University of Georgia, 30602 Athens, GA, USA; 8 Division of Sustainable Process Engineering, Luleå University of Technology, 97187 Luleå, Sweden

**Keywords:** Alkaline pretreatment, Oxidative delignification, Xylose fermentation

## Abstract

**Background:**

A two-stage chemical pretreatment of corn stover is investigated comprising an NaOH pre-extraction followed by an alkaline hydrogen peroxide (AHP) post-treatment. We propose that conventional one-stage AHP pretreatment can be improved using alkaline pre-extraction, which requires significantly less H_2_O_2_ and NaOH. To better understand the potential of this approach, this study investigates several components of this process including alkaline pre-extraction, alkaline and alkaline-oxidative post-treatment, fermentation, and the composition of alkali extracts.

**Results:**

Mild NaOH pre-extraction of corn stover uses less than 0.1 g NaOH per g corn stover at 80°C. The resulting substrates were highly digestible by cellulolytic enzymes at relatively low enzyme loadings and had a strong susceptibility to drying-induced hydrolysis yield losses. Alkaline pre-extraction was highly selective for lignin removal over xylan removal; xylan removal was relatively minimal (~20%). During alkaline pre-extraction, up to 0.10 g of alkali was consumed per g of corn stover. AHP post-treatment at low oxidant loading (25 mg H_2_O_2_ per g pre-extracted biomass) increased glucose hydrolysis yields by 5%, which approached near-theoretical yields. ELISA screening of alkali pre-extraction liquors and the AHP post-treatment liquors demonstrated that xyloglucan and β-glucans likely remained tightly bound in the biomass whereas the majority of the soluble polymeric xylans were glucurono (arabino) xylans and potentially homoxylans. Pectic polysaccharides were depleted in the AHP post-treatment liquor relative to the alkaline pre-extraction liquor. Because the already-low inhibitor content was further decreased in the alkaline pre-extraction, the hydrolysates generated by this two-stage pretreatment were highly fermentable by *Saccharomyces cerevisiae* strains that were metabolically engineered and evolved for xylose fermentation.

**Conclusions:**

This work demonstrates that this two-stage pretreatment process is well suited for converting lignocellulose to fermentable sugars and biofuels, such as ethanol. This approach achieved high enzymatic sugars yields from pretreated corn stover using substantially lower oxidant loadings than have been reported previously in the literature. This pretreatment approach allows for many possible process configurations involving novel alkali recovery approaches and novel uses of alkaline pre-extraction liquors. Further work is required to identify the most economical configuration, including process designs using techno-economic analysis and investigating processing strategies that economize water use.

## Introduction

Relative to liquid transportation-fuels derived from petroleum, biofuels derived from the pretreatment and enzymatic hydrolysis of lignocellulosic biomass offers many potential sustainability benefits. Many chemical pretreatments have been explored, covering a diverse range of pH, solvents, and temperature. They have a wide range of impacts on cell-wall polymers [1]. Alkaline hydrogen peroxide (AHP) pretreatment significantly improves the enzymatic digestibility of grasses (for example, corn stover and switchgrass) [2-4] because of several distinctive features of their cell walls [5]. Previous work has employed AHP as a single-step pretreatment and required more than 100 mg H_2_O_2_ per g biomass to improve digestibility over pretreatment with NaOH alone (unpublished data). The likely reason is that a significant portion of the H_2_O_2_ is presumably consumed by reacting with alkali-solubilized aromatics rather than with the more recalcitrant lignin remaining in the cell walls. Additionally, catalytic amounts of transition metals in the biomass may contribute to the non-productive disproportionation of H_2_O_2_. For these process configurations, H_2_O_2_ would be the primary cost input to the process and must be decreased. At 100 mg H_2_O_2_ per g biomass, the cost of H_2_O_2_ is $1.50 to $2.00 per gal of ethanol. New H_2_O_2_ production technology improvements may significantly decrease this cost; however to be economical, new pretreatment process configurations must be identified that use significantly less H_2_O_2_.

At mild temperatures (< 100°C), treatment of grasses with NaOH induces significant solubilization of xylan and lignin relative to dicots [6] and thus, has been proposed as a standalone pretreatment for grasses including corn stover [7], wheat straw [8], sweet sorghum [9], and switchgrass [10]. In addition, NaOH is proposed as a deacetylation step prior to dilute acid pretreatment of corn stover [11]. The current work investigates an improved AHP process by introducing an NaOH pre-extraction step prior to subsequent AHP delignification. This approach requires significantly less H_2_O_2_ (and NaOH) by first solubilizing and removing easily extracted lignin and xylan with alkali. Then, in a subsequent AHP step, an oxidizing post-treatment removes the more recalcitrant lignin from the cell walls. Additional advantages of a mild-temperature alkaline pre-extraction are that xylan degradation to saccharinic acids through alkaline peeling would not be substantial. This alkali-solubilized xylan can be potentially recovered and used in other applications [6,12]. Further, soluble inhibitors of both enzymes and microbes are removed using pre-extraction. This two-stage NaOH pretreatment approach provides many opportunities for integrating processes with the alkali pre-extraction liquor, including: (1) concentration in a multiple-effect evaporator, combustion in a traditional smelting black liquor recovery boiler, a fluidized bed boiler, or wet air oxidation [13,14], and alkali recovery through recausticization using a lime regeneration cycle or autocausticization using Na-borate [15] or Fe_2_O_3_[13] in the extraction liquor; (2) the use of gasification rather than combustion, which would enable the synthesis of Fischer-Tropsch, mixed alcohol, or dimethyl ether fuels from the sulfur-free syngas [16,17], or (3) recovery of alkali-solubilized lignin and xylan by ultrafiltration [18,19] or by acidification and filtration [20,21], which can produce a low-sulfur solid fuel or a feedstock for fuels, chemicals, and polymeric materials.

Alkaline pre-extraction is similar to soda pulping of graminaceous agricultural residues (for example, sugarcane bagasse, wheat straw, and rice straw) in which delignification by NaOH alone is followed by an oxidative delignification or bleaching stage [22]. These commercial pulping technologies are currently performed in China, India, South Africa, and Australia, among others [23,24]; however, there are different process objectives for a cellulosic biofuels process. Grasses have both lower lignin content and differences in their cell-wall structures; therefore, alkali pulping is performed at considerably milder conditions than for woody plants [25-27]. Atmospheric soda pulping of sugarcane bagasse at temperatures < 100°C has been proposed to have positive economic advantages for small-scale operations because of the lower capital requirements [28].

In the literature, there are many precedents for two-stage pretreatments employing an alkaline or oxidative post-treatment. As relevant examples, alkaline pretreatment/pulping followed by oxygen delignification has been applied to softwoods [29] and hardwoods [30], lime pretreatment followed by peracetic acid delignification has been applied to kenaf (an herbaceous dicot) [31], AHP delignification has been applied as a post-treatment coupled to dilute-acid [32] and liquid hot water pretreatment [4] for grasses such as corn stover and switchgrass, and NaOH delignification has been coupled to the autohydrolysis of corn stover [33]. To our knowledge, mild alkaline pretreatments of grasses coupled to oxidative post-treatments - comparable to the commercial practices of alkaline pulping and oxidative delignification or bleaching of non-wood feedstocks - have not been explored as pretreatments. There is substantial need to improve knowledge of processing conditions that optimize hydrolysis yields and minimize sugar degradation. With this in mind, the scope of the present work is to investigate conditions for alkaline pre-extraction of corn stover coupled to an oxidative or alkali-only post-treatment. Specifically, this work investigates: (1) the impact of NaOH loading and solids concentration on composition, biomass mass yields, and alkali consumption during alkaline pre-extraction; (2) improvement in glucose hydrolysis yield by subsequent AHP or post-treatment with NaOH alone; (3) the comparison of alkali-solubilized glycans during alkali pre-extraction and AHP post-treatment using an ELISA screen for non-cellulosic cell-wall glycans, and (4) the fermentability of the sugar hydrolysates generated by this two-stage pretreatment approach using *Saccharomyces cerevisiae* strains metabolically engineered and evolved for xylose fermentation.

## Results and discussion

### NaOH pre-extraction

**Figure 1:**
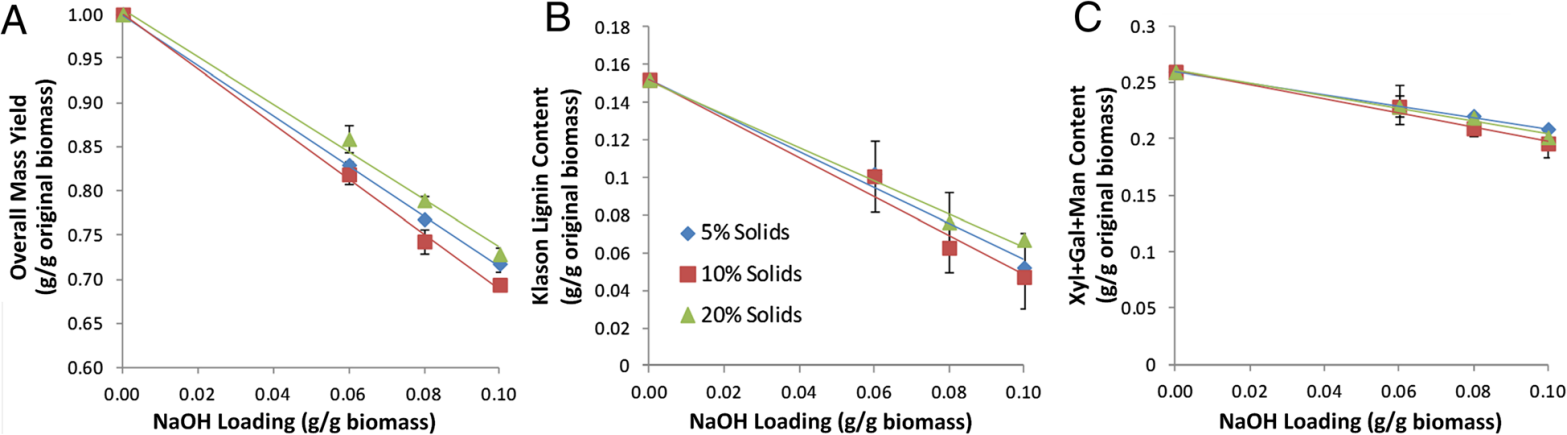
**Impact of NaOH and solids loading (w/v) during alkaline pre-extraction on the solubilization of cell-wall polymers and extractives.** Results are plotted for **(A)** total biomass solids, **(B)** Klason lignin, and **(C)** hemicelluloses (Xyl + Gal + Man). Pre-extraction was performed at 80°C for 1 h.

Treatment of graminaceous monocots such as corn stover with alkali at relatively modest concentrations and temperatures can solubilize up to 50% of the original biomass, primarily extractives, hemicelluloses (xylans), and lignin [6]. This ability to solubilize plant cell walls can be exploited by pretreatments that improve the enzymatic hydrolysis of cell-wall polysaccharides to fermentable sugars in biofuel processes. Figure 1 presents the relationship between mass loss and compositional change in the biomass as a function of alkaline pre-extraction conditions. The obvious trend is that increasing alkali loading during the pre-extraction process increases solubilization of hemicellulose (primarily xylan) and lignin. Glucan content exhibited a minor decrease (data not shown), which likely results from removing glucan-containing hemicelluloses as well as sucrose and glucose in the water-soluble extractives.

A relatively low alkali loading alkaline pre-extraction allows for several advantageous potential process outcomes, including highly selective lignin removal versus xylan. Further, it decreases alkali consumption and substantially decreases the required alkali recovery in the recausticization process, which decreases the capital requirements. Although lignin removal helps improve hydrolysis yields, xylan retention improves the overall sugar yields for the subsequent hydrolysis. In this sense, pre-extraction must balance lignin removal (to improve the enzymatic hydrolysis) with xylan retention (to improve sugar hydrolysis yields). At relatively mild alkali concentrations, the maximum xylan removals were only 15 to 24% (Figure 1C). Across all extraction conditions, the average selectivity is 1.6 g lignin removed per g xylan removed. Earlier work for switchgrass demonstrated that under comparable extraction conditions with increasing alkali far above the conditions used in the present work, the xylan extractability reached a plateau at 70% removal [6].

**Figure 2:**
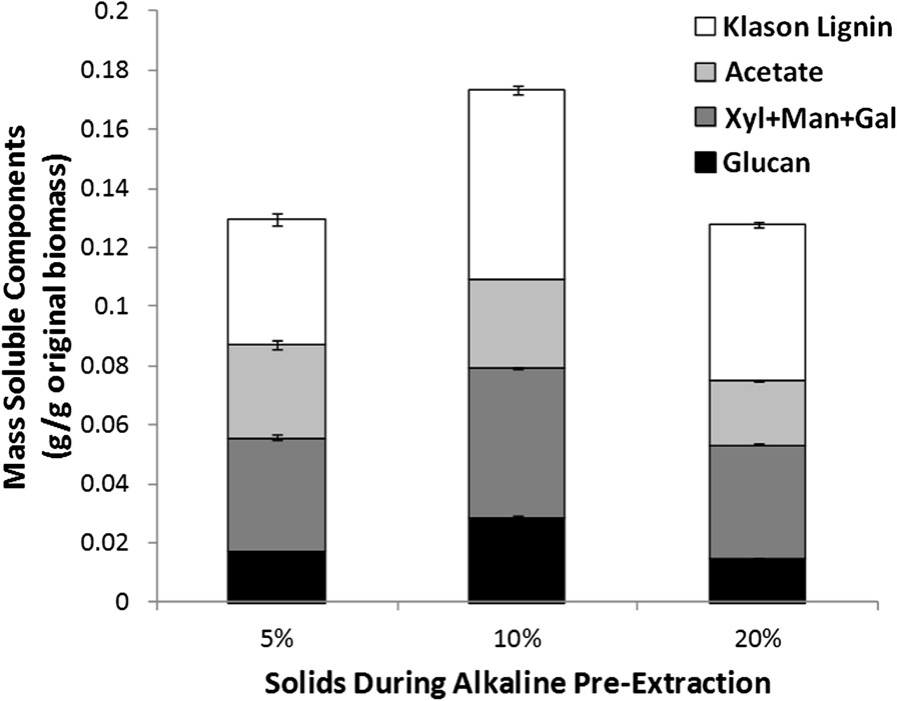
**Content of lignin, acetate, and total polymeric and oligomeric neutral polysaccharides in alkaline pre-extraction liquors as a function of solids loading during alkaline pre-extraction.** Pre-extraction was performed at 0.08 g NaOH per g corn stover, at 80°C for 1 h and results show a maximum extraction of cell wall biopolymers at 10% (w/v) solids, which is likely a consequence of imperfect alkali penetration into the biomass at 20% (w/v) solids because of the limitations of the laboratory-scale mixing.

Operating biomass conversion processes at high solids concentrations minimizes process water-use and reduces costs for energy, capital equipment, and product recovery [34-36]. During pre-extraction, solids concentration is important because it impacts the pH for comparable alkali loadings. For example, an alkali loading of 0.10 g NaOH per g biomass is an alkali concentration of only 5 g/L at 5% (w/v) biomass solids concentration and 20 g/L at 20% (w/v) solids, resulting in substantial differences in pH. In contrast, alkaline pulping using Kraft pulping for woody biomass and soda pulping for grasses may use alkali concentrations in the range of 150 to 180 g/L corresponding to alkali loadings of approximately 1.0 g NaOH per g biomass [14] and mass yields of approximately 50%. As a function of solids concentration, Figure 1A shows that alkaline pre-extractions at 20% (w/v) solids concentration are slightly less effective than at 10% (w/v) solids. This is counter-intuitive because higher solids concentrations should yield higher pH values for the same alkali loading and presumably result in more extraction. However, a problem with high-solids treatment is the difficulty of penetrating alkali into the biomass because of limitations of laboratory mixing. Consistent with these results is the concentration of solubilized cell-wall biopolymers in the pre-extraction liquor (Figure 2). As the solids increase from 5 to 10% (w/v), extraction yields for hemicelluloses (primarily xylan) and lignin, increase and then decrease at 20% (w/v). Interestingly, the estimated acetate yields from the 5, 10, and 20% (w/v) solids alkaline pre-extraction were 96, 96, and 67%, respectively. Again, the failure to completely deacetylate the biomass indicates that alkali did not perfectly impregnate the biomass because of poor mixing. Suitable mixing/impregnation would likely eliminate this inefficiency. Previous pre-extractions [8% (w/v) solids, 0.048 g/g NaOH loading, 70°C, 2 h] removed 30 to 45% of acetyl groups from corn stover [11].

**Figure 3:**
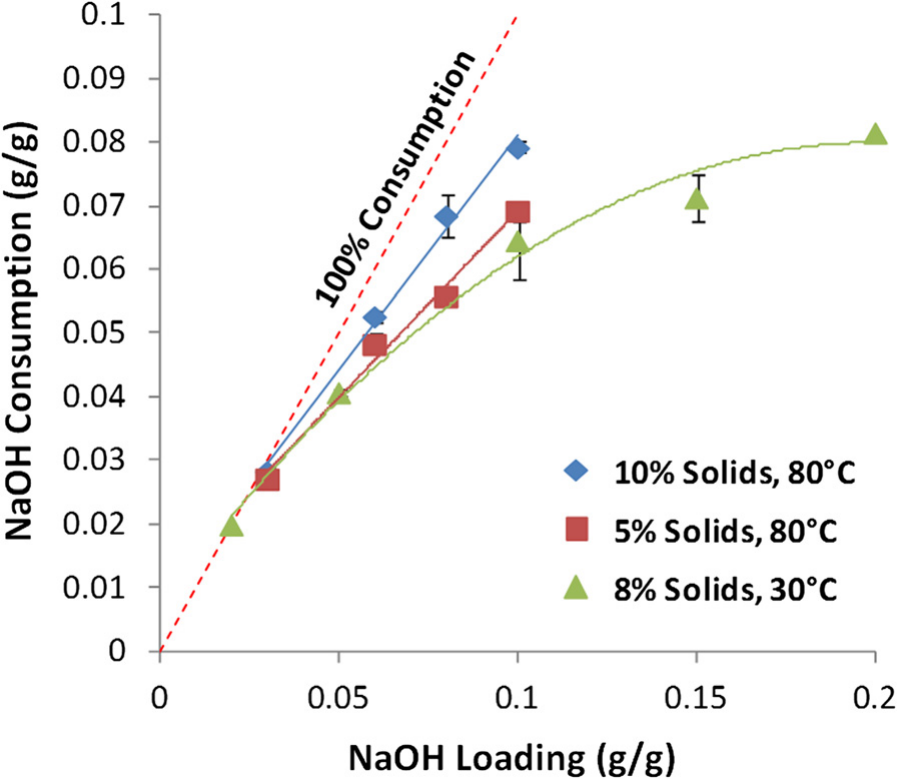
**Alkali consumption during alkaline pre-extraction of corn stover as a function of solids concentration (w/v), NaOH loading, and temperature.** The alkali consumption is shown to approach a horizontal asymptote at between 0.08 and 0.10 g/g NaOH loading.

Alkali is consumed in both saponification reactions (for example, triglycerides and tannins and compounds acylated to hemicelluloses and lignins such as acetyl, *p*-coumaryl, and feruloyl esters) and potentially by carboxylic acid degradation products of lignin and polysaccharides (for example, formic acid, saccharinic acids, hydroxy acids). Figure 3 plots alkali consumptions for a range of pre-extraction conditions. At 80˚C and higher solids concentrations (10%, w/v), alkali consumption is nearly complete for the conditions tested in this work, although slightly less alkali is consumed at 5% (w/v) solids concentration. Alkali consumption profiles were also generated for extractions at 30˚C over a wider range of NaOH loadings. Using this wider range of NaOH loadings, alkali consumption approaches a maximum of 0.09 to 0.10 g NaOH per g biomass. Previously published results for mild-temperature NaOH pretreatment of wheat straw found comparable alkali consumptions [8].

### AHP and alkali-only post-treatment

**Figure 4:**
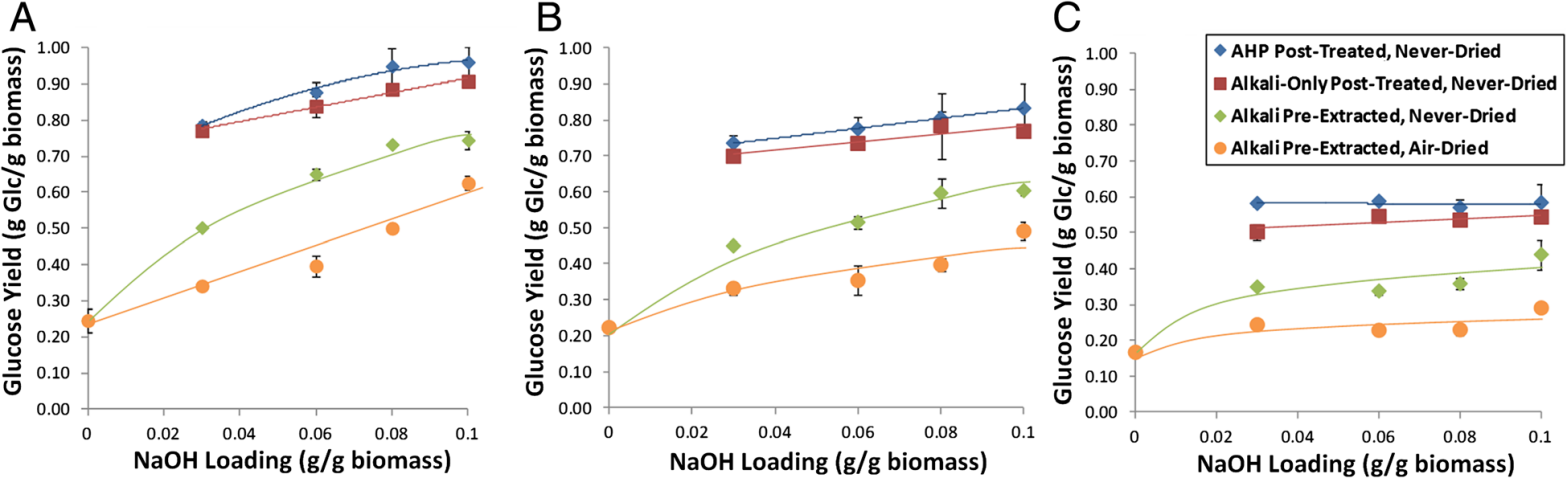
**Hydrolysis yields for alkali-extracted corn stover as a function of alkaline pre-extraction condition.** Post-treatment was performed and 25 mg H_2_O_2_ /g biomass, **(A)** 15 mg protein/g glucan enzyme loading, **(B)** 10 mg protein/g glucan enzyme loading, and **(C)** 5 mg protein/g glucan enzyme loading. Extraction was performed at 10% (w/v) solids and 80°C for 1 h with 100% displacement of pre-extraction liquor with distilled water following pre-extraction.

Alkali pre-extracted corn stover was generated for a range of alkali loadings. First, corn stover was washed to remove all of the solubles from the pre-extraction and subjected to either an AHP post-treatment (25 mg H_2_O_2_ per g biomass loading, pH of 11.5, 30˚C, 24 h) or a subsequent alkali post-treatment (pH 11.5, NaOH only). Following pH neutralization with concentrated H_2_SO_4_ with no additional solid–liquid separation or washing, these treated samples were subjected to 24 h of hydrolysis using a commercial cellulase cocktail. Figure 4 presents the hydrolysis yields of both air-dried and never-dried alkali pre-extracted corn stover. Several notable results can be observed that merit comment.

The hydrolysis yields were high for short hydrolysis times (24 h) and low enzyme loadings (5 to 15 mg protein per g glucan). Although these enzyme loadings are low compared to others reported for lignocellulose hydrolysis, they are still an order of magnitude higher than the amylase enzyme loadings required for starch hydrolysis in the corn ethanol industry [37]. Only 24 h is employed for hydrolysis; therefore, longer hydrolysis times would result in higher sugar yields, particularly for low enzyme loadings. Another obvious result is the substantial difference between the hydrolysis yields for air-dried versus never-dried alkali pre-extracted corn stover. Air-drying delignified corn stover decreases glucose hydrolysis yields by 10 to 25%. This results from drying-induced hornification or irreversible pore collapse of cell walls at the nanometer scale and potentially the collapse of the entire lumen at the whole-cell scale [38]. Pore collapse is a well-established outcome of drying delignified plant cell walls; therefore, pore properties are a strong function of biomass history (drying, pressing, storage). This phenomena, which is well-known from literature on pulp and paper, decreases water penetration in cell-wall pores [38], which decreases cellulase penetration and hydrolysis [39]. Because delignified cell walls are highly susceptible to drying-induced pore collapse, no drying was performed between alkali pre-extraction, AHP post-treatment, and hydrolysis.

Next, an additional post-treatment with either alkali only or AHP at relatively modest H_2_O_2_ loading (25 mg H_2_O_2_ per g biomass) was performed to assess the impact on enzymatic hydrolysis yields (Figure 4). The highest achievable glucose hydrolysis yields were obtained for alkali pre-extracted corn stover (0.08 and 0.10 g NaOH/g biomass) subjected to AHP post-treatment. Figure 4 shows that at an enzyme loading of 15 mg protein per g glucan, 24-h hydrolysis yields reached 95 to 96%. Monomeric xylose yields for hydrolysis only (for example, not including xylan lost during prior treatments) were 50 to 60% for both AHP and alkali post-treated samples (data not shown), although these should increase if hydrolysis times are extended. Although the glucose yields were statistically higher than alkali-only post-treated materials (*P* > 0.999), identifying that the alkali post-treatment alone can yield a material that is highly susceptible to hydrolysis is an important finding. These alkali-only post-treated materials exhibited 24-h glucose hydrolysis yields that were, on average, only 5% lower than the AHP post-treated yields. Considering the cost of H_2_O_2_ (approximately $700 per tonne), the 5% increased hydrolysis yields corresponds to an increase in the estimated overall ethanol yield (assuming 0.45 g/g and 0.30 g/g yields of ethanol from glucose and xylose, respectively) from approximately 58 gal/tonne to 62 gal/tonne. This additional cost for H_2_O_2_ corresponds to $2.50 for each marginal gallon of EtOH generated, or alternatively $0.17 per gallon of ethanol overall.

**Figure 5:**
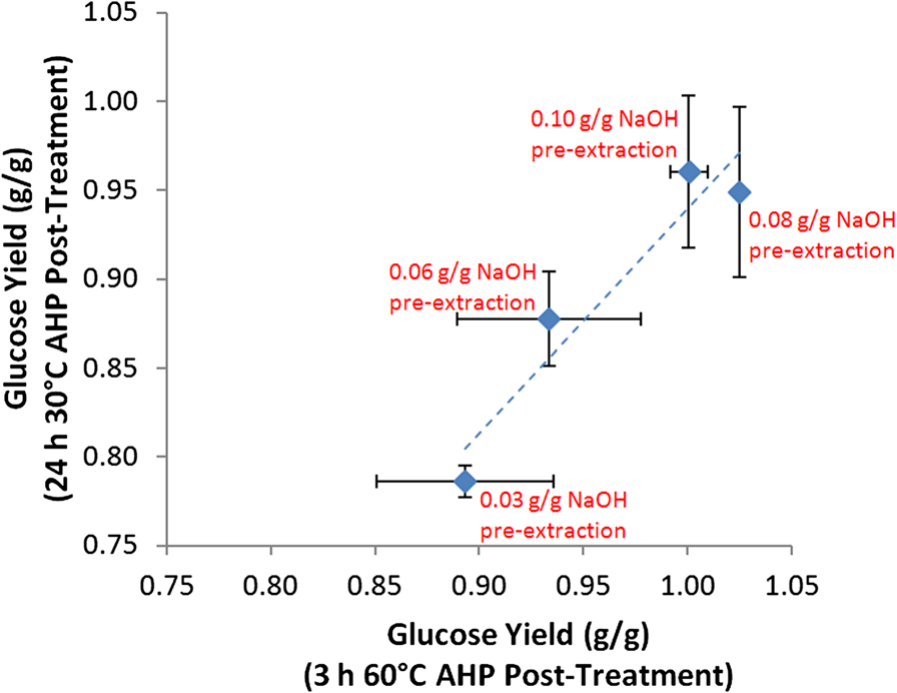
Impact of shorter time, higher temperature post-treatment on glucose hydrolysis yields for a range of alkali pre-extraction conditions.

Shorter time, higher temperature AHP post-treatments were also tested (60˚C for 3 h) for some conditions that would be more realistic for a process employing this post-treatment. Compared to the 24 h, 30˚C treatments, the yields were comparable or slightly higher (Figure 5), indicating that these conditions would be preferable.

### ELISA screening of alkali pre-extraction and AHP post-treatment liquors for solubilized cell-wall glycans

**Figure 6:**
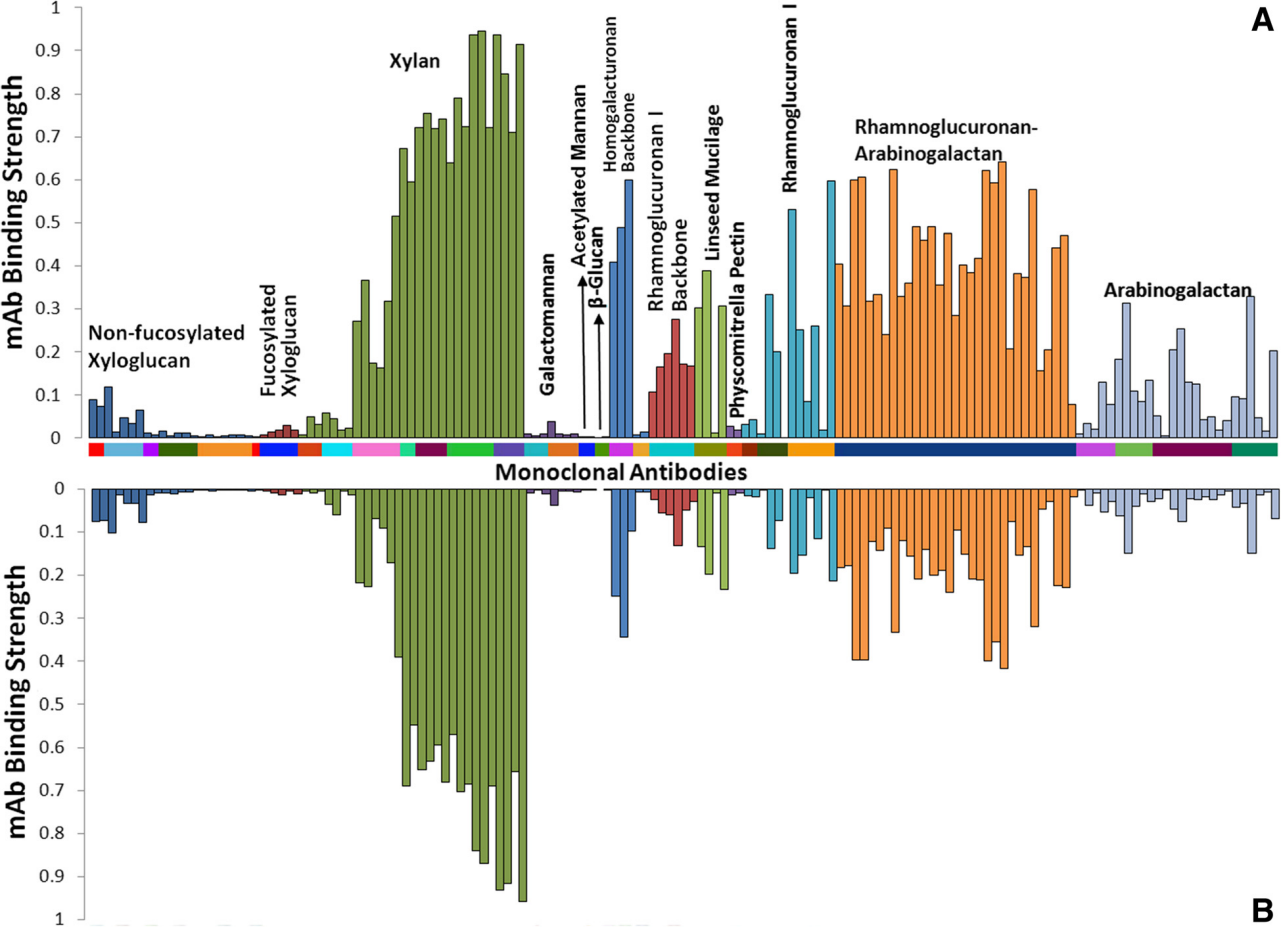
**ELISA screening of glycans solubilized in pre- and post-treatment liquors using a panel of cell wall glycan-directed monoclonal antibodies (mAbs).** These results plot the mAb binding affinity for liquors of **(A)** alkali pre-extracted corn stover at 0.10 g/g and **(B)** alkaline hydrogen peroxide (AHP) post-treatment of this pre-extracted corn stover with the mAb categories defined in Additional file 1.

In the analyses of major non-cellulosic plant glycans in several bioenergy crops including corn stover, currently available collections of cell wall glycan-directed monoclonal antibodies (mAbs) have been instrumental. Earlier studies employing ELISA screens of these mAbs against diverse structurally characterized plant cell-wall glycans have categorized these mAbs into multiple groups based on their specificity to distinct cell-wall glycans [40,41]. Taking advantage of this, ELISA screens with cell wall glycan-directed mAbs were performed to determine the range and relative abundance of the non-cellulosic polysaccharides that were solubilized during alkaline pre-extraction, and how their distribution and relative abundance are altered in the AHP post-treatment liquor. Xylan epitopes represent the most abundant recognizable hemicellulose epitopes in both the pre-extraction liquor (Figure 6A) and the AHP post-treatment liquor (Figure 6B). This is indicated by the significant binding of xylan-3, xylan-4, and xylan-5 groups of mAbs (Additional file 1) that had been previously shown to be specific to either unsubstituted (homoxylans) or highly substituted xylans (glucurono(arabino)xylans) [40]. Other hemicellulosic epitopes, such as those for non-fucosylated xyloglucan and fucosylated xyloglucan, were present only in trace abundance, indicating that xyloglucans are not solubilized during these processes and that the majority of the solubilized xylose in Figure 2 arises from glucuronoxylans rather than from xyloglucans. Among these trace amounts of xyloglucans, the presence of non-fucosylated xyloglucan epitopes were in relatively higher proportions compared to fucosylated xyloglucan epitopes. The relative distribution of solubilized hemicelluloses (xylans and trace amounts of xyloglucans) is reasonably constant between the alkaline pre-extraction liquor and the AHP post-treatment liquor, indicating that both treatments solubilize similar pools of hemicellulosic glycans.

According to Figure 2, 7.6% of the original glucan in the biomass is solubilized in the pre-extraction liquor and can be hypothesized to be hemicelluloses (for example, extracted β-glucans, xyloglucans, and glucomannans) and/or water-soluble sucrose, monomeric glucose, and potentially even phenolic glycosides [42]. β-glucans are known to be important components of the primary cell walls of graminaceous monocots and are hypothesized to play the role of xyloglucans in dicots [43]. Interestingly, this work shows that β-glucans, which are known to be present in cell walls of corn stover, are not present in either the alkaline pre-extraction or the AHP post-treatment liquors. It is important to note that ELISA screens using mAbs conducted here allow the detection of relatively larger cell-wall glycans that effectively adsorb to ELISA plates. Information on small glycan molecules (for example, oligomeric glycans, sucrose, and monosaccharides) is lost in the ELISA screen analyses, because these small molecules do not adhere to the plates and thus cannot be detected by mAbs [40].

Other major glycan epitopes present in both alkali-solubilized and AHP post-treatment liquors were pectic backbones (as indicated by the binding of HG backbone-1 and RG-I backbone-1 groups of mAbs), pectic arabinogalactan (as indicated by the binding of RG-I/AG groups of mAbs), and arabinogalactan (as indicated by the binding of AG-1 through AG-4 groups of mAbs) epitopes. These epitopes for pectic arabinogalactan polysaccharides relative to the total glycan abundance in the extract is notably decreased in the AHP post-treatment liquor, because it is likely that a higher fraction of these more extractable glycans are removed in the pre-extraction.

### Generation of high-sugar corn stover hydrolysates

**Figure 7:**
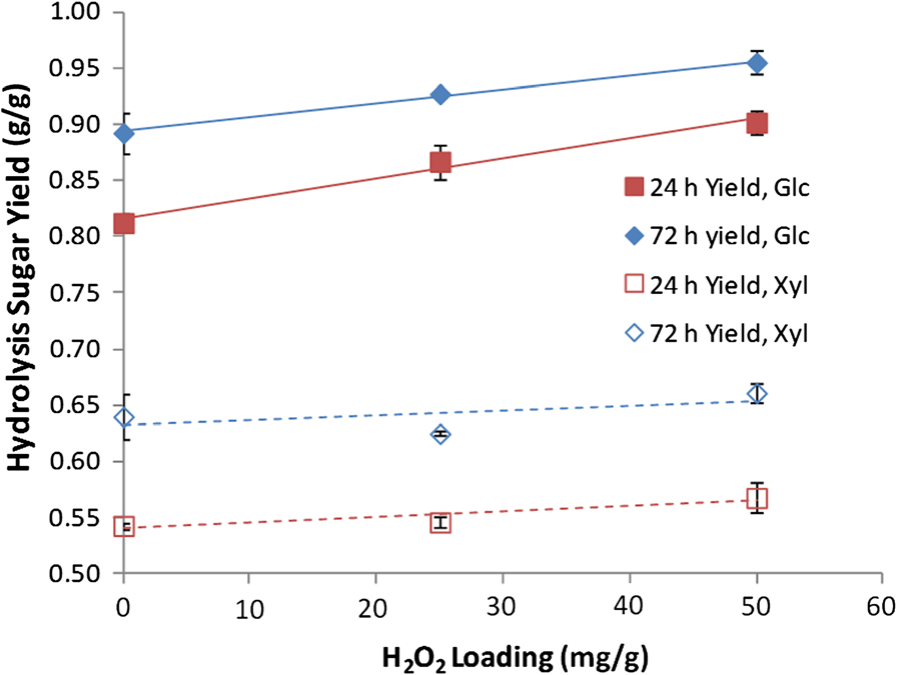
**Effect of H**_**2**_**O**_**2 **_**loading during alkaline hydrogen peroxide (AHP) post-treatment on the hydrolysis yields of alkaline pre-extracted, never-dried corn stover.** Alkaline pre-extraction was performed at 10% (w/v) solids with a 70% displacement of the pre-extraction liquor prior to AHP post-treatment at 23.5% (w/v) solids. Hydrolysis was performed at 10% (w/v) solids.

Next, hydrolysates were generated for fermentation using slightly different conditions than were used in the preliminary screening of conditions for pre-extraction and post-treatment (Figure 4). Specifically, pre-extracted corn stover (0.08 g NaOH/g biomass) was not washed, but only subjected to dewatering before being subjected to AHP post-treatment. For these hydrolysates only 70% of the pre-extraction liquor was removed and replaced with water up to the same solids content, resulting in a displacement ratio of 0.70. For this process, multistage counter current washing schemes could be envisioned that makes efficient use of water and result in substantially more alkali recovery from the pre-extracted biomass [44]. The 24-h hydrolysis yields for these incompletely washed materials are presented as a function of H_2_O_2_ loading (Figure 7) with 0 mg/g H_2_O_2_ loading representing alkali-only post-treatment at pH 11.5. From these data, the AHP post-treatment clearly improves the subsequent enzymatic hydrolysis for glucose, whereas the improvement realized for xylose hydrolysis yields are minimal. Additionally, the results show only slightly lower hydrolysis yields than the thoroughly washed material at comparable treatment conditions (Figure 4A). This indicates that it is likely that residual solubles (for example, xylan and soluble aromatics from the alkaline pre-extraction) slightly inhibit the hydrolysis, which has been clearly demonstrated in the past [45,46]. This approach resulted in minimal xylan degradation, with only 5% of the total intial xylan unaccounted for in a material balance across solid and liquid phases (data not shown). For the condition of 25 mg H_2_O_2_ per g biomass in Figure 7, alkaline pre-extraction removed 58% of the lignin and the subsequent AHP post-treatment resulted in a total of 73% lignin removal (data not shown).

Using this approach with incomplete washing, hydrolysates of alkali-pre-extracted corn stover subjected to AHP post-treatment were generated. Specifically, the hydrolysis performed at two different solids concentrations during the pre-extraction, post-treatment, and hydrolysis generate sugars at different concentrations. Table 1 summarizes the conditions used to generate these hydrolysates as well as hydrolysate sugar and quantified compounds that are known to inhibit fermentation rates. To minimize capital costs and separation costs for ethanol recovery, high ethanol titers (requiring high sugar titers) and high ethanol productivities are necessary. As an example, corn ethanol fermentations often achieve ethanol concentrations in excess of 18% (v/v) [37]. As such, it would be advantageous to generate high sugar titers in lignocellulose hydrolysates produced from high-solids enzymatic hydrolysis. A drawback to the fermentation of high sugar titer lignocellulose hydrolysates is that inhibitors deriving from the degradation and modification of cell-wall polymers during pretreatment are typically present that are toxic to fermentation.

**Table 1:** Conditions used to generate corn stover hydrolysates and the sugar and inhibitor concentration of these hydrolysates

	**Hydrolysate 1**	**Hydrolysate 2**
Solids to NaOH pre-extraction (w/v)	10.0%	20.0%
Solids to AHP post-treatment (w/v)	10.0%	23.5%
Enzyme cocktail (protein mass ratio)	CTec2 + HTec2 (0.77:0.23)	CTec2 + HTec2 (0.77:0.23)
Enzyme loading (mg/g glucan)	15	15
Glc (g/L)	51.4	74.6
Xyl (g/L)	20.9	37.9
Formate (g/L)	0	0
Acetate (g/L)	0.28	0.37

AHP, alkaline hydrogen peroxide.

Our previous work has demonstrated that hydrolysates generated from one-stage AHP pretreatment of corn stover and switchgrass are already highly fermentable without detoxification with xylose-fermenting *Saccharomyces* strains used in our laboratory [47]. For a process using an alkaline pre-extraction, the alkali-solubilized xylan, lignin, extractives, and alkali-saponifiable compounds including acetate, *p*-coumarate, and ferulate as well as inorganics (Na^+^) are removed prior to hydrolysis in the pre-extraction liquor. In particular, Na^+^ in these hydrolysates is expected to be at several-fold lower concentrations (approximately 100 to 200 mM) than those presented in our previous work [47]. As a consequence, it is expected that hydrolysates generated using this approach will be substantially less inhibitory to fermentation. This is comparable to the alkaline deacetylation pre-extraction performed at the National Renewable Energy Laboratory (NREL) prior to dilute-acid pretreatment, which generated hydrolysates substantially less inhibitory to fermentation by metabolically engineered *Zymomonas mobilis*[11].

### Hydrolysate fermentation by xylose-fermenting yeast strains

**Figure 8:**
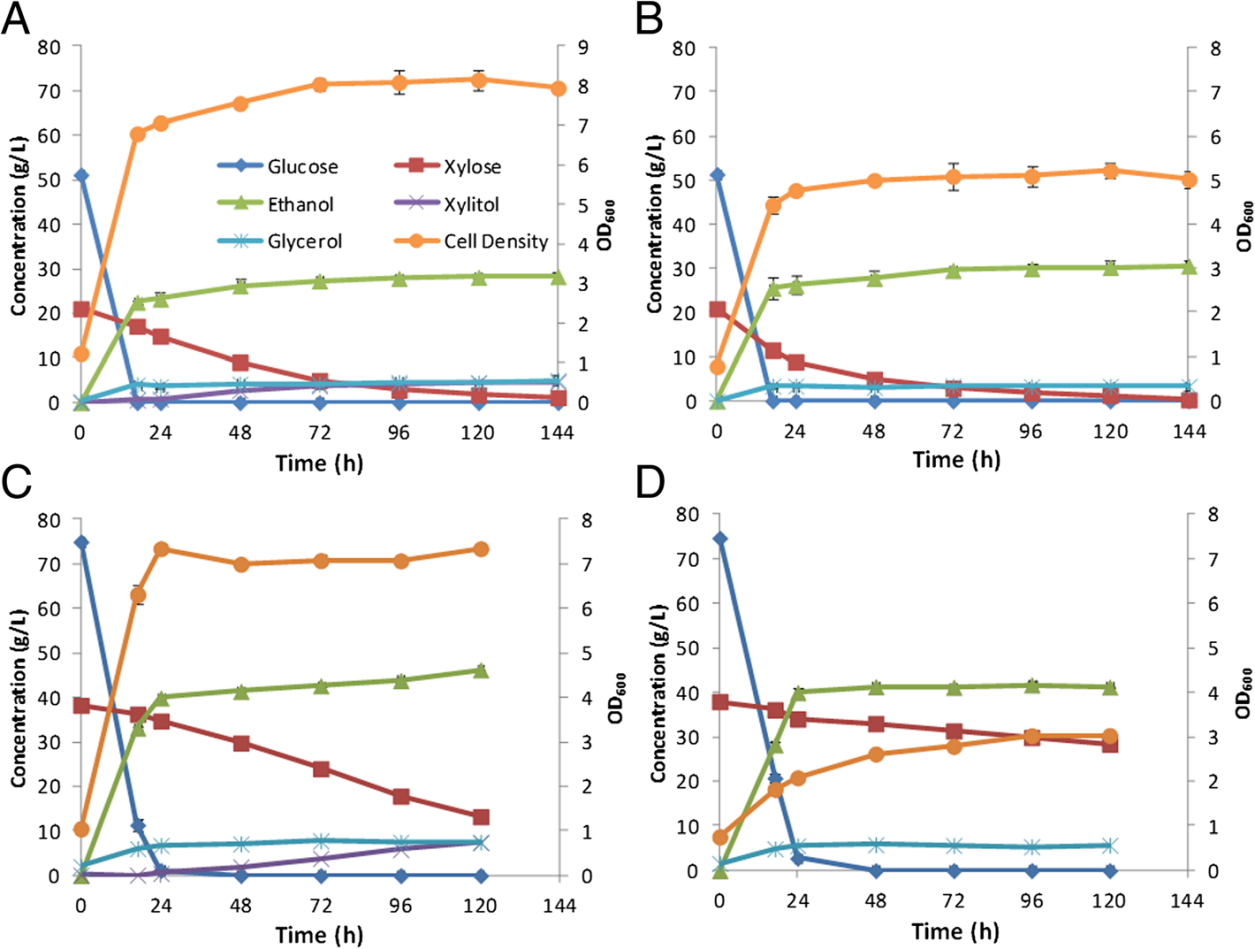
**Hydrolysate fermentation kinetics.** These represent fermentation of hydrolysates of alkali pre-extracted, alkaline hydrogen peroxide (AHP) post-treated corn stover by *Saccharomyces cerevisiae* strains metabolically engineered for xylose fermentation using either the xylose reductase (XR) + xyitol dehydrogenase (XDH) pathway (strain Y73) or the xylose isomerase (XI) pathway (strain Y128). Conditions include **(A)** Y73 in low-sugar hydrolysate, **(B)** Y128 in low-sugar hydrolysate, **(C)** Y73 in high-sugar hydrolysate, **(D)** Y128 in high-sugar hydrolysate.

**Figure 9:**
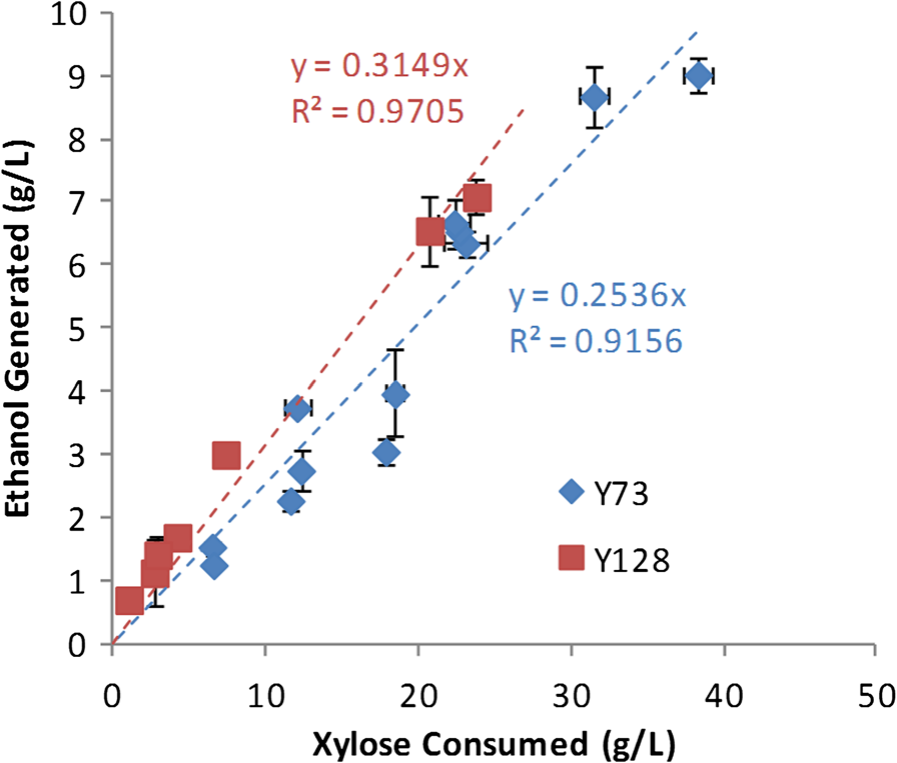
**Determination of ethanol yields in strains Y73 and Y128.** Plotting xylose consumption versus ethanol production for both corn stover hydrolysates (Table 1 and Figure 7) for strains Y73 and Y128 results in the regression of the slope that represents the average ethanol yield on xylose, *Y*_*EtOH/Xyl*_, (g/g). The average ethanol yield on xylose in the strain utilizing the xylose isomerase pathway (Y128) was slightly higher than that of the strain utilizing the reductase/xylitol dehydrogenase pathway (Y73).

To demonstrate the fermentability of these hydrolysates, two hydrolysates were next subjected to fermentation by evolved, metabolically engineered *S. cerevisiae* strains. The two strains include strain Y73, which was engineered to assimilate xylose using xylose reductase (XR) + xylitol dehydrogenase (XDH) and strain Y128 which expresses a bacterial xylose isomerase (XI) to facilitate xylose conversion to xylulose and subsequently to ethanol. Figure 8 presents the fermentation kinetics for these two hydrolysates by these two strains. For the low-sugar-concentration corn stover hydrolysate (Hydrolysate 1) complete conversion of both glucose (51 g/L) and xylose (21 g/L) to ethanol was realized within 100 h. For both strains, the glucose was rapidly fermented within 18 h, whereas xylose was fermented more rapidly in strain Y128 (Figure 8B). The high-sugar hydrolysate fermentations (Hydrolysate 2) resulted in incomplete xylose consumption after 120 h for both strains (Figures 8C and D) with ethanol titers reaching more than 45 g/L for strain Y73. The average ethanol yields (*Y*_*EtOH/Xyl*_) for each strain can be estimated by generating a regression for a plot of xylose consumption versus ethanol generation (Figure 9) and these were found to be 0.31 g/g for strain Y128 and 0.25 g/g for strain Y73. Strain Y128, which performed better for the low-sugar hydrolysate, showed slower xylose consumption for the high-sugar hydrolysate.

The fermentation results for strain Y73 are comparable to the performance on one-stage AHP-pretreated corn stover hydrolysates of similar sugar concentrations, which in our previously work [47] required nearly 240 h to completely consume all the sugars and generate more than 40 g/L ethanol. Rather than inhibition by compounds in the hydrolysate, this slower xylose consumption is likely a consequence of combined stresses from the long fermentation time, which includes ethanol inhibition and nutrient depletion, because only minimal media components were added to the hydrolysate. Overall, these results provide positive validation that these high-sugar hydrolysates are highly fermentable to ethanol because of the low concentration of inhibitors present.

## Conclusions

This work supports a number of notable conclusions that have important implications for cellulosic biofuels processes that use NaOH pretreatment. Alkaline pre-extraction coupled to alkaline or alkaline-oxidative post-treatment of corn stover can yield a biomass that is highly digestible at relatively low enzyme loadings and was highly fermentable because soluble inhibitors to both enzymes (aromatics and xylan oligomers) and microbes (*p*-hydroxycinnamic acids, acetate, and most of the Na^+^) are removed during the pre-extraction. Minimal β-glucan and xyloglucan were identified in any of the liquors indicating that these cell-wall glycans are more tightly bound into the cell walls and are not likely to be strong contributors to the alkaline pre-extraction or AHP post-treatment xylan or glucan content. Pre-extraction at the high 20% (w/v) solids concentration resulted in less efficient treatment than at 10% (w/v) solids, presumably because of limitations associated with laboratory-scale mixing. The application of low loadings of H_2_O_2_ (25 mg/g) as a delignifying agent following the alkaline pre-extraction improved hydrolysis yields by 5% on average relative to post-treatment with alkali alone. In future work, the oxidant cost relative to the improved yield needs to be evaluated by applying techno-economic modeling, process design, and process optimization. Because water use is an important environmental component of cellulosic biofuels processes, additional ongoing work is focused on identifying process options that can economize water through minimization and recovery. This includes the use of process liquors as backset while increasing the solids content during processing and/or increasing the washing efficiency to decrease the energy load to the evaporators prior to chemical recovery.

## Methods

### Biomass

Corn stover (*Zea mays* L. Pioneer hybrid 36H56) was the same batch of material reported previously [5] and was milled (Circ-U-Flow model 18-7-300, Schutte-Buffalo Hammermill, LLC, Buffalo, NY, USA) to pass a 5-mm screen. Moisture content, structural carbohydrates including glucan, xylan + mannan + galactan, acid-soluble lignin and acid-insoluble lignin of corn stover before and after pretreatment as well as alkali-solubilized polysaccharides and lignin were determined according to NREL standardized analytical procedures (NREL/TP-510-42618; NREL/TP-510-42619; NREL/TP-510-42623) with modifications as reported previously [5,6].

### NaOH pre-extraction and AHP post-treatment

Alkali pre-extracted corn stover was prepared by soaking corn stover in NaOH solution at 80°C for 1 h. The solids loading of corn stover during alkaline pre-extraction was 5, 8, 10, or 20% (w/v) with NaOH loadings based on the mass of corn stover. For some cases, after pre-extraction the remaining insoluble solids were filtered and washed with excess deionized water to remove all solubles. A sample of the wet biomass cake was taken to determine moisture content after pre-extraction. For materials using incomplete washing, after extraction the liquid was manually squeezed out of the biomass using a filtration cloth (Miricloth, EMD Millipore, Billerica, MA, USA). Subsequently, a known quantity of the removed liquor was added back to the filter cake to yield a 70% removal of the liquor. For AHP or alkali-only post-treatment, the experiments were conducted at 30°C for 24 h in 250-mL shake flasks, in which H_2_O_2_ was mixed with 6 g alkali-extracted corn stover. The H_2_O_2_ loading was 25 mg H_2_O_2_ per g biomass for AHP post-treatment, whereas no H_2_O_2_ was included in post-treatment with alkali alone. The pH was periodically adjusted to 11.5 using 5 M NaOH. Short-time, high-temperature AHP post-treatment was performed at 60°C for 3 h. Biomass mass yields after alkaline pre-extraction were determined gravimetrically by performing the pre-extraction on 1 g corn stover, washing out solubles using several cycles of centrifugation, decanting, and resuspension in distilled water. The washed pre-extracted biomass pellet was oven-dried at 105°C to determine the mass yield. Consumption of NaOH was quantified by titrating diluted pre-extraction liquors to their equivalence point using 0.1 M HCl (DMS Titrino716, Metrohm, Herisau, Switzerland).

### Enzymatic hydrolysis

Hydrolysis was conducted at 50°C in a temperature-controlled incubator with orbital shaking at 160 rpm. Hydrolysis was performed at 10% (w/v) solids with the exception of hydrolysates used for fermentation (Hydrolysate 1 and Hydrolysate 2, Table 1) which were hydrolyzed at the same solids as AHP post-treatment with dilution only for pH adjustment. The enzyme cocktails Cellic CTec2 and HTec2 were provided by Novozymes A/S (Bagsværd, Denmark) with the protein content determined by the Bradford assay (Sigma-Aldrich, St. Louis, MO, USA) using bovine serum albumin as standard. Na-citrate buffer (0.05 M, pH 5.2) was used for alkali pre-extracted washed material, whereas incompletely washed whole slurries of alkali pre-extracted or AHP post-treated corn stover were neutralized to pH 5.2 [48] using 72% (w/w) H_2_SO_4_ (approximately 3.3 mg H_2_SO_2_/g biomass). Cellic CTec2 and HTec2 were added at 15 mg total protein content per g glucan in biomass. The protein mass ratio of CTec2:HTec2 was 0.77:0.23 based on the protein content according to the Bradford assay (Sigma-Aldrich, St. Louis, MO, USA). Hydrolysis yields for saccharification were determined according to our previously outlined methodology [36] based on the experimentally determined composition after pre-extraction.

### Hydrolysate fermentation

*S. cerevisiae* strains engineered for xylose fermentation to ethanol using either XR + XDH (strain Y73) as reported earlier [47] or the XI (strain Y128) were supplied by Trey Sato (Great Lakes Bioenergy Research Center, University of Wisconsin, Madison, WI, USA). For fermentation of the hydrolysates, the whole hydrolysis slurry was centrifuged (16,000 × g) and the supernatant was decanted. Yeast nitrogen base (YNB) without amino acids and ammonium sulfate and urea were added to the supernatant to final concentrations of 1.67 g/L and 2.27 g/L, respectively. The pH was next adjusted to pH 5.5 using NaOH pellets and the hydrolysate was filter sterilized (Millipore Stericup, Billerica, MA, USA) prior to inoculation. Fermentations were performed in 250-mL shake flasks capped with fermentation locks (Bacchus & Barleycorn Ltd. Shawnee, KS, USA) with a working volume of 50 mL at 30°C and agitation in a rotary incubator at 180 rpm. Yeast seed cultures were prepared in yeast extract peptone dextrose media as described previously [47]. A known volume of the seed culture at a known optical density (OD)_600_ was centrifuged and resusupended in hydrolysate to yield an initial OD_600_ of 1.0. Following inoculation and sampling, flasks were purged with nitrogen to maintain anaerobic conditions.

### Analysis of hydrolysates and fermentations

The concentrations of glucose, xylose, and ethanol in hydrolysate and fermentation samples were determined by high-performance liquid chromatography (HPLC) (Agilent 1100 Series, Agilent Technologies, Santa Clara, CA, USA) using an Aminex HPX-87H column (Bio-Rad Laboratories, Inc., Hercules, CA, USA) operating at 65°C, a mobile phase of 0.005 M H_2_SO_4_, a flow rate of 0.6 mL/minute, and detection by refractive index. Cell densities (OD_600_) were determined spectrophotometrically (Biomate 3, Thermo-Fisher Scientific, Waltham, MA, USA) following a 10-fold dilution in water. All plotted data points represent averages of sample duplicates at a minimum, whereas error bars represent the data range.

### ELISA screening with a panel of cell wall-directed monoclonal antibodies

To conduct the ELISA screens with a comprehensive collection of cell wall glycan-directed mAbs, each liquor sample was loaded onto the ELISA plates (Corning 384-well clear flat-bottom polystyrene high-bind microplate, product #3700) on an equal carbohydrate basis (15 μL per well from a solution of 20 μg/mL carbohydrate) for conducting ELISA screens as described previously [40,41]. Plant glycan-directed mAbs were from laboratory stocks (CCRC, JIM and MAC series) at the Complex Carbohydrate Research Center (available through CarboSource Services; http://www.carbosource.net) or were obtained from BioSupplies (Bundoora, Australia) (BG1, LAMP). A description of the mAbs used in this study can be found in Additional file 1 which includes links to a web database, Wall*Mab*DB (http://www.wallmabdb.net) that provides detailed information about each antibody.

## Abbreviations

AHP: alkaline hydrogen peroxide; ELISA: enzyme-linked immunosorbent assay; mAbs: monoclonal antibodies; NREL: National renewable energy laboratory; OD: optical density; XDH: xyitol dehydrogenase; XI: xylose isomerase; XR: xylose reductase; YNB: yeast nitrogen base.

## Competing interests

The authors declare that they have no competing interests.

## Authors’ contributions

DBH: conception and design, data collection and analysis, manuscript writing, critical revision and final approval of the manuscript. TL: conception and design, data collection and analysis, manuscript writing, critical revision, and final approval of the manuscript. DLW: data collection and analysis, manuscript writing, and final approval of the manuscript. SP: data collection and analysis, manuscript writing, and final approval of the manuscript. ML: data collection and analysis, manuscript writing, and final approval of the manuscript. MGH: data analysis, critical revision, and final approval of the manuscript. All authors read and approved the final manuscript.

## Supplementary Material

Additional file 1 **Listing of plant cell wall glycan-directed monoclonal antibodies (mAbs) used for ELISA screening (Figure**6**).** The groupings of antibodies are based on a hierarchical clustering of ELISA data generated from a screen of all monoclonal antibodies (mAbs) against a panel of plant polysaccharide preparations that groups the mAbs according to the predominant polysaccharides that they recognize. The majority of listings link to the Wall*Mab*DB plant cell-wall monoclonal antibody database (http://www.wallmabdb.net) that provides detailed descriptions of each mAb, including immunogen, antibody isotype, epitope structure (to the extent known), supplier information, and related literature citations. Click here for file
