# Supplementary material for: Coupling alkaline pre-extraction with alkaline-oxidative post-treatment of corn stover to enhance enzymatic hydrolysis and fermentability
Source: Biotechnol Biofuels. 2014 Apr 3;7:48. doi: 10.1186/1754-6834-7-48 (PMC3997815; doi:10.1186/1754-6834-7-48)
Supplement: Additional file 1 — Listing of plant cell wall glycan-directed monoclonal antibodies (mAbs) used for ELISA screening (Figure6). The groupings of antibodies are based on a hierarchical clustering of ELISA data generated from a screen of all monoclonal antibodies (mAbs) against a panel of plant polysaccharide preparations that groups the mAbs according to the predominant polysaccharides that they recognize. The majority of listings link to the WallMabDB plant cell-wall monoclonal antibody database (http://www.wallmabdb.net) that provides detailed descriptions of each mAb, including immunogen, antibody isotype, epitope structure (to the extent known), supplier information, and related literature citations. [file 1754-6834-7-48-S1.pdf]

Additional File 1: List of plant cell wall glycan-directed monoclonal antibodies (mAbs) used for ELISA screening analyses (Figure 6). The groupings of antibodies are based on a hierarchical clustering of ELISA data generated from a screen of all mAbs against a panel of plant polysaccharide preparations[1,2] that groups the mAbs according to the predominant polysaccharides that they recognize. The majority of listings link to the Wall*Mab*DB plant cell wall monoclonal antibody database (http://www.wallmabdb.net) that provides detailed descriptions of each mAb, including immunogen, antibody isotype, epitope structure (to the extent known), supplier information, and related literature citations.

Glycan Group Recognized	mAb Names
Non-Fucosylated Xyloglucan-1	CCRC-M95<br>CCRC-M101
Non-Fucosylated Xyloglucan-2	CCRC-M104<br>CCRC-M89<br>CCRC-M93<br>CCRC-M87<br>CCRC-M88
Non-Fucosylated Xyloglucan-3	CCRC-M100<br>CCRC-M103
Non-Fucosylated Xyloglucan-4	CCRC-M58<br>CCRC-M86<br>CCRC-M55<br>CCRC-M52<br>CCRC-M99
Non-Fucosylated Xyloglucan-5	CCRC-M54<br>CCRC-M48<br>CCRC-M49<br>CCRC-M96<br>CCRC-M50<br>CCRC-M51<br>CCRC-M53
Non-Fucosylated Xyloglucan-6	CCRC-M57
Fucosylated Xyloglucan	CCRC-M102<br>CCRC-M39<br>CCRC-M106<br>CCRC-M84<br>CCRC-M1
Xylan-1/XG	CCRC-M111<br>CCRC-M108<br>CCRC-M109
Xylan-2	CCRC-M119<br>CCRC-M115

	CCRC-M110<br>CCRC-M105
Xylan-3	CCRC-M117<br>CCRC-M113<br>CCRC-M120<br>CCRC-M118<br>CCRC-M116<br>CCRC-M114
Xylan-4	CCRC-M154<br>CCRC-M150
Xylan-5	CCRC-M144<br>CCRC-M146<br>CCRC-M145<br>CCRC-M155
Xylan-6	CCRC-M153<br>CCRC-M151<br>CCRC-M148<br>CCRC-M140<br>CCRC-M139<br>CCRC-M138
Xylan-7	CCRC-M160<br>CCRC-M137<br>CCRC-M152<br>CCRC-M149
Galactomannan-1	CCRC-M75<br>CCRC-M70<br>CCRC-M74
Galactomannan-2	CCRC-M166<br>CCRC-M168<br>CCRC-M174<br>CCRC-M175
Acetylated Mannan	CCRC-M169<br>CCRC-M170
β-Glucan	LAMP<br>BG1
HG Backbone-1	CCRC-M131<br>CCRC-M38<br>JIM5
HG Backbone-2	JIM136<br>JIM7

RG-I Backbone	CCRC-M69<br>CCRC-M35<br>CCRC-M36<br>CCRC-M14<br>CCRC-M129<br>CCRC-M72
Linseed Mucilage RG-I	JIM3<br>CCRC-M40<br>CCRC-M161<br>CCRC-M164
Physcomitrella Pectin	CCRC-M98<br>CCRC-M94
RG-Ia	CCRC-M5<br>CCRC-M2
RG-Ib	JIM137<br>JIM101<br>CCRC-M61<br>CCRC-M30
RG-Ic	CCRC-M23<br>CCRC-M17<br>CCRC-M19<br>CCRC-M18<br>CCRC-M56<br>CCRC-M16
RG-I/Arabinogalactan	CCRC-M60<br>CCRC-M41<br>CCRC-M80<br>CCRC-M79<br>CCRC-M44<br>CCRC-M33<br>CCRC-M32<br>CCRC-M13<br>CCRC-M42<br>CCRC-M24<br>CCRC-M12<br>CCRC-M7<br>CCRC-M77<br>CCRC-M25<br>CCRC-M9<br>CCRC-M128<br>CCRC-M126<br>CCRC-M134

	CCRC-M125<br>CCRC-M123<br>CCRC-M122<br>CCRC-M121<br>CCRC-M112<br>CCRC-M21<br>JIM131<br>CCRC-M22<br>JIM132<br>JIM1<br>CCRC-M15<br>CCRC-M8<br>JIM16
Arabinogalactan-1	JIM93<br>JIM94<br>JIM11<br>MAC204<br>JIM20
Arabinogalactan-2	JIM14<br>JIM19<br>JIM12<br>CCRC-M133<br>CCRC-M107
Arabinogalactan-3	JIM4<br>CCRC-M31<br>JIM17<br>CCRC-M26<br>JIM15<br>JIM8<br>CCRC-M85<br>CCRC-M81<br>MAC266<br>PN 16.4B4
Arabinogalactan-4	MAC207<br>JIM133<br>JIM13<br>CCRC-M92<br>CCRC-M91<br>CCRC-M78
Unidentified	MAC265<br>CCRC-M97

References:

1. Pattathil S, Avci U, Baldwin D et al. A comprehensive toolkit of plant cell wall glycan-directed monoclonal antibodies. *Plant Physiol* 2010; 153:514-525.

2. Pattathil S, Avci U, Hahn MG Immunological approaches to plant cell wall and biomass characterization: glycome profiling. *Methods Mol Biol* 2012, 908: 61-72.
